# Quantification of DAS VSP Quality: SNR vs. Log-Based Metrics

**DOI:** 10.3390/s22031027

**Published:** 2022-01-28

**Authors:** Aleksei Titov, Vladimir Kazei, Ali AlDawood, Ezzedeen Alfataierge, Andrey Bakulin, Konstantin Osypov

**Affiliations:** 1 Department of Geophysics, Colorado School of Mines, Golden, CO 80401, USA; 2 Aramco Americas—Houston Research Center, Houston, TX 77084, USA; kosypov@gmail.com; 3 EXPEC Advanced Research Center, Dhahran 34465, Saudi Arabia; ali.dawood.18@aramco.com (A.A.); ezzedeen.alfataierge@aramco.com (E.A.); andrey.bakulin@aramco.com (A.B.)

**Keywords:** distributed acoustic sensing (DAS), vertical seismic profiling (VSP), data metrics

## Abstract

The initial quantification of data quality is an important step in seismic data acquisition design, including the choice of sensing strategy. The signal-to-noise ratio (SNR) often drives the choice of distributed acoustic sensing (DAS) parameters in vertical seismic profiling (VSP). We compare this established approach for data quality assessment with metrics comparing DAS data products to available well logs. First, we create kinematic and dynamic data products derived from original seismic data, such as the interval velocity and amplitude of P-wave arrivals. Next, we quantify the quality of derived data products using well log data by calculating various statistical metrics. Using a large dataset of 220 different VSP experiments with a fixed source location and various DAS acquisition parameters, such as gauge length (GL), conveyance type, and lead-in length, we analyzed the statistical distribution of various metrics. The results indicate the decoupling between seismic-based and log-based metrics as well as between the quality of dynamic and kinematic data-products for the same record. Therefore, we propose using fit-for-purpose metrics to optimize the acquisition cost. In particular, for ray-based tomographic processing, it is sufficient to use traveltime-based metrics. On the other hand, for advanced dynamic analysis, amplitude-based metrics define the quality of final processing products. Hence, it is crucial to use fit-for-purpose metrics to optimize DAS VSP acquisition.

## 1. Introduction

Vertical seismic profiling (VSP) is a geophysical method for estimating the elastic properties of subsurface layers using seismic waves generated by a source located at the surface and receivers located in a borehole. The main benefits of VSP surveys in comparison with surface seismic (where both sources and receivers are placed on the surface) are the use of direct waves and the proximity of the receivers to the region of interest [1]. A seismic vibrator, a weight drop, dynamite, and various types of air guns can be used as sources in VSP surveys. The generated waves are recorded into a receiver array installed in a well. Conventionally, electrical sensors, such as geophones and hydrophones, are used for capturing downhole vibrations. Spacing between geophones (channel spacing) is usually more than 10 m (for some applications could be less), the length of the array is limited by a few hundred meters, and the tool is typically moved 6–8 times to cover the entire length of the well, which can be logistically difficult. To overcome these limitations, VSP acquisition based on distributed acoustic sensing (DAS) [2,3,4] is emerging as an alternative technology to acquire borehole seismic data. Moreover, DAS VSP can be acquired in cases when the conventional acquisition is very challenging or even not possible, e.g., in subsea umbilical cables [5] or during well stimulation [6,7]. DAS utilizes conventional telecommunication or special engineered fibers and turns them into a dense array of strain, strain-rate, or motion sensors [8]. The interrogator unit generates a laser pulse (or pulses) which is scattered by natural or induced density impurities inside the fiber cable. DAS utilizes an elastic Rayleigh scattering mode to monitor local displacement variations along the fiber [9]. DAS covers all the length of the well with very small (about 1 m, for GL of 10 m) channel spacing [10,11], which prevents spatial aliasing in seismic records and reduces acquisition costs.

To make an educated decision on the acquisition design for a particular subsurface environment, one should quantify the quality of the acquired data. Various approaches exist to calculate the most used for this purpose signal-to-noise ratio (SNR). The calculation can be performed in the time or frequency domain, and various calculation windows can be chosen, which affects the final metrics. The SNR calculation does not require any additional measurements and can be directly applied to raw VSP data.

The quality of VSP data impacts the value of information recorded in the dataset and is a function of the subsurface geology and acquisition parameters. While we cannot modify the subsurface geology, we do have control over the parameters of the source (e.g., effort, type, location, number of shot repetitions, etc.) and the receiver (e.g., type, location, etc.). When DAS is used as a receiver, additional decisions need to be made on DAS acquisition parameters, such as gauge length (GL), fiber and conveyance type, a distance of lead-in fiber, etc. The GL is the length of optical fiber over which the recorded signal is optically averaged [8]. The spatial resolution is directly proportional to GL, while the optical signal-to-noise ratio (SNR) is inversely proportional to it [12]. Related seismic SNR is also inversely proportional to the GL if the GL is smaller than half of the seismic wavelength [12,13]. Usually, single-mode telecommunication grade fibers are used for DAS acquisition, while some systems are capable of interrogating a multimode fiber (that is typically used for distributed temperature sensing). With advances in DAS VSP, the development of an engineered fiber [14] has become another option, enabling higher optical SNR for better data quality. For the conveyance type, we can distinguish between five primary types: (i) cemented behind the casing, (ii) clamped to the tubing, (iii) wireline or carbon rod installation, (iv) inserted into a capillary tubing (also called control line) and (v) bare fiber installation. The quality of seismic data varies due to different types of installation [15]. The lead-in distance also has a significant impact on data quality [5,16].

The data-driven SNR metrics might not fully describe the quality of the data. Another way to quantify seismic data quality is to validate the quality of derived data products by comparing them with other available information, such as well logs. This paper presents a comparative analysis of data-based and log-based metrics for DAS VSP quality assessment.

## 2. Data Acquisition and Analysis

### 2.1. Data Acquisition

**Figure 1:**
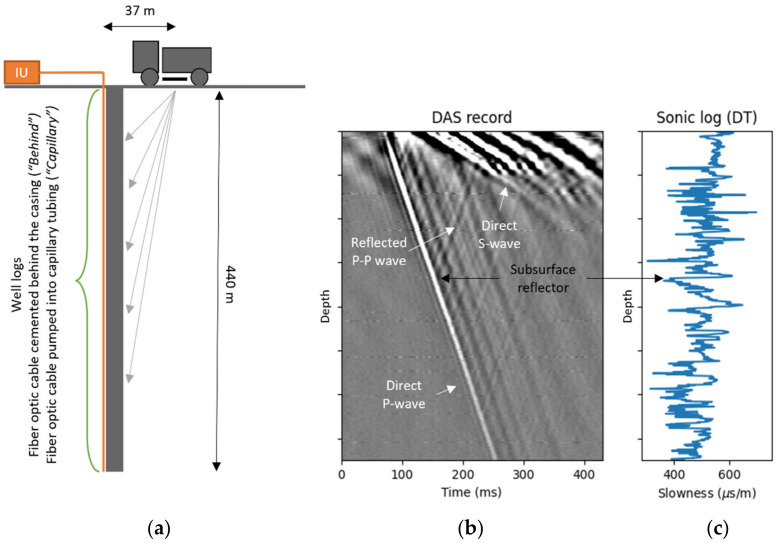
(**a**) Schematic representing survey geometry. Vibroseis truck located 37 m away from the wellhead generated seismic waves captured by fiber-optic cables installed along ~440 m well interrogated by an interrogator unit (IU) shown in orange. (**b**) Example of seismic data recorded by DAS after correlation with vibroseis sweep. Direct P-wave, direct S-wave, and example of one of the reflected P-P wave arrivals are indicated. (**c**) The compressional sonic log data in terms of slowness as measured by the sonic logging tool. Note that the vertical scale is the same for all subplots.

DAS VSP data were acquired using a shallow (~440 m) land test well in Houston, which is used as a test site to evaluate current DAS VSP technology and develop quality assessment techniques before field deployment. The well is completed with a combination of steel and fiberglass and provides environments for conducting both cased-hole and open-hole measurements [16]. A detailed description of the test facility is presented in [13,16,17]. A schematic representing the survey geometry is shown in Figure 1a. A vibroseis truck generates seismic waves with sweep frequencies from $f_{s}$ = 8 Hz to $f_{e}$ = 120 Hz at a distance of 37 m away from the wellhead. The seismic waves propagate in the subsurface and are recorded by fiber cables installed inside the wellbore. We use the data acquired by the cemented behind the casing (for shortness will call it “behind”) telecommunication cable and the engineered cable installed into the capillary tubing (for shortness, we will name it “capillary”). The capillary tube is also cemented behind the casing allowing fibers to be easily pumped down, evaluated, and retrieved [16]. Data and data acquisition parameters are summarized in Table 1.

An interrogator unit (IU) located on the surface sends light pulses down the fiber cable to capture vibrations along the axis of fiber with a 1 m spatial and 1 ms temporal sampling rate. An example of a seismic wavefield recorded by DAS after correlation with the vibroseis pilot sweep is shown in Figure 1b. The vertical axis represents the depth of the DAS channel and the horizontal axis the time after the shot. Typical features include a direct compressional or P-wave, a direct shear or S-wave, and reflected P-P waves, highlighted in the figure. The reflected waves originate from subsurface reflectors when a direct P-wave strikes them. In this work, we focus on data products extracted from the direct arrival of the P-wave, namely, the interval velocity and P-wave amplitude.

We used compressional sonic log data (Figure 1c), to calculate log-based metrics for DAS data quality evaluation. The sonic log was acquired with a major vendor sonic tool with the standard 15.24 cm depth sampling. Note that the subsurface reflectors visible in the seismic data (Figure 1b) match the rapid changes in slowness (Figure 1c).

### 2.2. Processing Workflow

**Figure 2:**
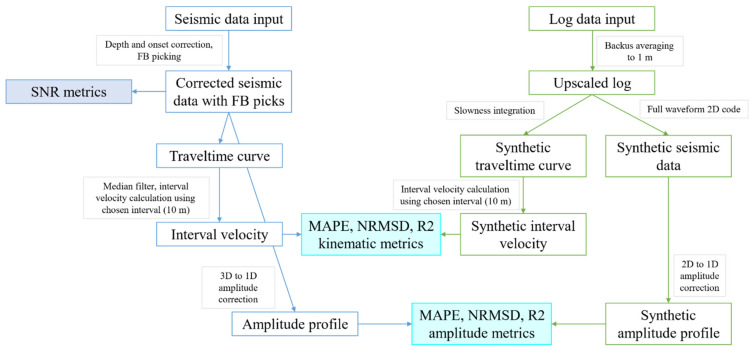
Data analysis workflow. DAS VSP seismic data and log data are inputs. The blue branch shows seismic data processing, while the green branch indicates operations with log data to generate “true” references for quantitative evaluation of DAS VSP data quality.

The data analysis workflow is shown in Figure 2. The blue branch shows seismic data processing, while the green branch indicates operations applied to the well log. Seismic data analysis starts with the correction of depth (channel mapping) and time (source onset). After that, first-breaks (FB), corresponding to the peak in direct P-wave arrival, are picked for all channels. Using FB picks, various SNR metrics are calculated. Next, the median filter with kernel 3 is applied to the traveltime curve, and an interval velocity over 10 m (scale hyperparameter) is computed using cosine correction for the source offset (37 m). Simultaneously, the 1D amplitude profile (corrected for geometrical spreading) is extracted by multiplying field amplitudes by receiver depth.

As the first step of well log data processing, it is upscaled to 1 m using Backus averaging. Next, the synthetic traveltime curve is calculated by integrating an upscaled slowness curve. The interval velocity is calculated using the same approach as for seismic data, including the median filtering of synthetic FB picks (no offset correction is needed in this case). The upscaled log is also used for full wavefield DAS modeling using a 2D elastic pseudospectral modeling code [18] with a wavelet extracted from the field data using a source matching algorithm [19]. The amplitudes from the synthetic seismogram are extracted in a similar way as for the field data. The 1D amplitude profile is derived by multiplying the synthetic amplitude by the square root of the synthetic receiver depth. Note that the correction is different from the field case scenario as the modeling was conducted in 2D. Next, the log-based kinematic and dynamics metrics are calculated. They include the mean absolute percentage error (MAPE), the normalized root-mean-square deviation (NRMSD), and the R2 score [20], which we define in the next section (Equations (3)–(5)).

MAPE reflects an average absolute error in the estimates. NRMSD is essentially the standard deviation of the estimate from the true value normalized to the true value itself. The R2 score reflects the part of variation that inversion can fit correctly. If the prediction is worse than the mean value, then R2 can be negative.

### 2.3. Calculated Metrics

#### 2.3.1. Data-Based SNR Metrics

There are two main approaches for the calculation of SNR in VSP data. First is a time-domain approach, based on root-mean-square (RMS) values for signal and noise in the determined windows. After that, the SNR can be calculated in terms of decibels (dB). For amplitude SNR can be expressed as:

(1) $$\mathrm{SNR}=20\log\frac{{\mathrm{RMS}}_{\mathrm{signal}}}{{\mathrm{RMS}}_{\mathrm{noise}}}.$$

**Figure 3:**
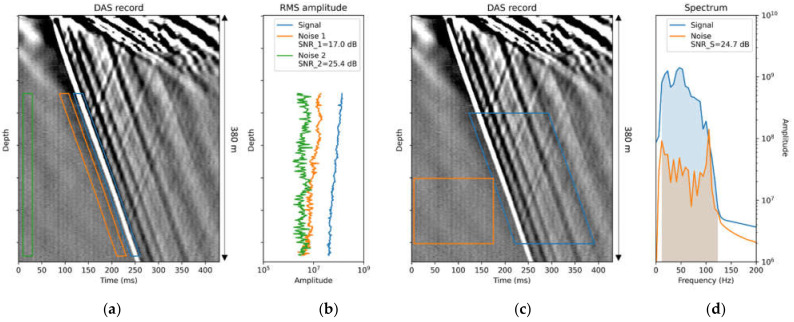
SNR calculation procedures. (**a**) DAS record with indicated time-domain signal (blue), noise 1 (orange), and noise 2 (green) windows for time-domain RMS calculation. (**b**) RMS amplitudes for windows shown in the previous panel. Time-domain SNR values for various noise windows (SNR_1, SNR_2) are shown. (**c**) DAS record with indicated signal (blue) and noise (orange) windows for frequency-domain RMS calculation. (**d**) Signal and noise spectra. Spectral SNR_S is shown.

While it is standard for the signal window to choose a 20 ms window (which equals to a dominant wave period) centered around direct P-wave arrival (FB) [14,21], the procedure of selecting a noise window varies. E.g., [21] selecting the window right before the P-wave arrival, while [14] choosing the noise window at the beginning of the record. These two approaches usually lead to different SNR values. As for the former case, the noise can be influenced by sidelobes of the correlation with the sweep procedure. Figure 3a,b demonstrates the SNR calculation procedure in the time domain. The signal window with a derived RMS signal value is shown in blue, while the same for different noises are shown in orange and green. Note that the RMS of noise from the beginning of the record is much lower than the noise right before the first arrival, which leads to a significant difference in resulting SNR values. This example showcases that in each case, one should report the exact procedure on SNR calculations to make the results reproducible and comparable between various studies and research groups.

Another approach, e.g., [16], is based on a spectral method to calculate SNR. In this approach, first, the spectral amplitudes both for signal and noise are calculated. Selected windows are longer in time as more time samples are needed in a comparison, with the previous approach applying Fourier transform. Second, the spectral amplitudes are integrated over sweep frequencies (shaded areas). Finally, the SNR is calculated:

(2) $$\mathrm{SNR}\_S=20\log\frac{\int_{f_{s}}^{f_{e}}A_{signal}\left(f\right)df}{\int_{f_{s}}^{f_{e}}A_{noise}\left(f\right)df},$$

where $f_{s}$ = 8 Hz and $f_{e}$ = 120 Hz in our case. If the same time windows are used and the bandwidth for the spectral calculation is the same, then the definition following Equation (2) is similar to replacing the RMS with the mean absolute value in Equation (1). We apply the spectral approach to our example DAS record and show results in Figure 3c,d.

#### 2.3.2. Data Product Log-Based MAPE, NRMSD, R2 Metrics

**Figure 4:**
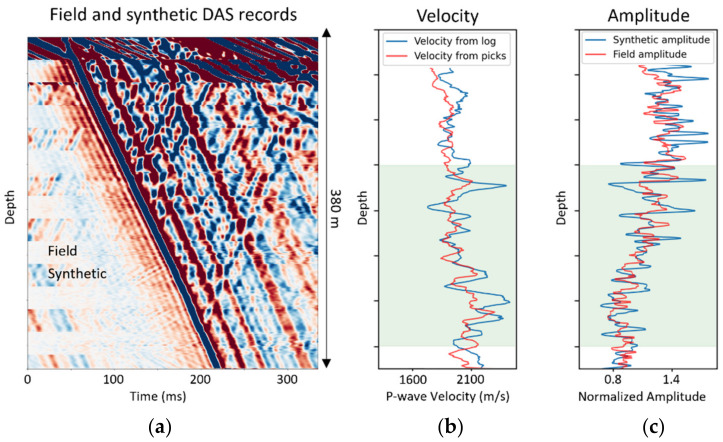
Log and seismic extracted data products. (**a**) Interleaved field and synthetic DAS records. (**b**) Velocity profile extracted from log (blue) and seismic (red). (**c**) Amplitude profile extracted from log (blue) and seismic (red). The green shaded area corresponds to the depth range where the metrics are calculated.

Examples of the extracted data products from the log, field and synthetic seismic data are shown in Figure 4, as was explained in the processing workflow (Figure 2). Note that field data for this example does not match the data presented in Figure 3. In the deeper part of the well, synthetic data are nearly indistinguishable from the field ones, which we illustrate by interleaving stripes of the data.

Next, the following metrics are used for comparison of velocities and amplitudes:

(3) $$\mathrm{MAPE}\left(m_{estimate},m_{true}\right)\equiv \frac{100\%}{n}\sum_{i}\left|\frac{m_{estimate,i}-m_{true,i}}{m_{true,i}}\right|,$$

(4) $$\mathrm{NRMSD}\left(m_{estimate},m_{true}\right)\equiv \frac{100\%\|m_{estimate}-m_{true}\|}{\left\|m_{true}\right\|},$$

(5) $$R2\left(m_{estimate},m_{true}\right)\equiv 1-\frac{\|m_{estimate}-m_{true}\|{}^{2}}{\|m_{true}-{\bar{m}}_{true}\|{}^{2}},$$

where $m_{estimate}$ is the estimated vector value derived from DAS VSP seismic data (shown in red in Figure 4b,c) and $m_{true}$ is the true value corresponding to the log-derived interval velocity or amplitude (shown in blue in Figure 4b,c). n is the length of the vector m, $m_{i}$ is the i-th element and the overbar averages elements of the corresponding vector. The inputs for the metrics calculation were extracted from seismic and log data, following the algorithm shown in Figure 2. The metrics were calculated for the depth interval from 200 m to 400 m, as the interval corresponds both to reliable seismic and well log data. For the example presented in Figure 4, kinematic (velocity) based metrics are as follows: MAPE_k = 5.09, NRMSD_k = 6.58, R2_k = 0.24. Amplitude-based metrics are MAPE_a = 11.14, NRMSD_a = 13.85, R2_a = 0.47.

## 3. Results

### 3.1. Statistical Analysis of the Metrics

**Figure 5:**
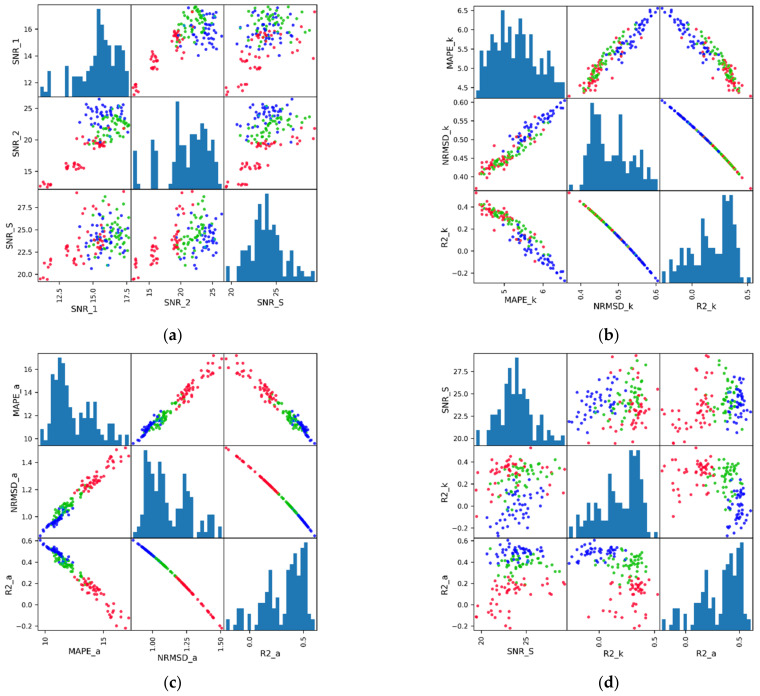
Scatter plot matrices for (**a**) data-based SNR metrics, (**b**) kinematic, (**c**) dynamic, and (**d**) combination of metrics. Each subplot shows the distribution of a particular metric and cross-plot between two metrics. The markers are color-coded accordingly to GL used for acquisition (red for 4 m, green for 8 m, and blue for 16 m).

We have calculated data-based and log-based metrics for 220 DAS VSP records acquired into fiber optic cables installed in our test well. To analyze how various metrics are related to each other and how their distributions are different, we used matrix plots, as shown in Figure 5. We used a subset of 144 records for this figure, which represented only behind the casing type of installation with GL of 4 m, 8 m, and 16 m. Note that we used all five lead-in configurations (from 0.04 km to 7.03 km). The diagonal plots of each subplot show the histogram representation of the particular metric distribution. Off-diagonal scattered cross-plots illustrate the relation between two selected metrics. Each dot is color-coded, according to the GL used for acquisition (red for 4 m, green for 8 m, and blue for 16 m).

Figure 5a shows the scatter plot matrices for the data-based SNR metrics, calculated in the time domain (SNR_1 and SNR_2) and frequency domain (spectral SNR_S) using windows for signal and noise shown in Figure 3 and Equations (1) and (2). A higher SNR represents better data quality. We can see that the distributions for SNR_1 and SNR_2 are similar, and some correlations between the metrics can be observed from the cross plots. Note that the quantitative assessment of correlation between various metrics, using the Pearson correlation coefficient, will be presented at the end of this section. We also see that the mean value is different (about 16 dB for SNR_1 and 24 dB for SNR_2). According to [12], the larger GL (up to half of the seismic wavelength) gives a better SNR. In our case the predominant wavelength is about 40 m, hence we should see an increase of the SNR up to 20 m GL. However, some experiments for GL = 8 m provide better results for the SNR_1, which can also be associated with different lead-in lengths in the considered dataset [16]. Besides the fact that the mean value for the spectral SNR_S is about the same as for the SNR_2, the shape of the distribution is different from other SNR metrics. There is no significant correlation between the SNR_S and other SNR metrics. It could be related to different windows chosen for the metrics calculations, as well as only the frequencies corresponding to the frequencies of the sources that are used. The larger GL also gives a higher SNR in the case of the SNR_S; however, the results for 8 m and 16 m are sometimes close to each other, which is similar to results presented in [16]. Again, we should stress the that it depends on the seismic wavelength.

Figure 5b shows the distributions and cross plots for kinematic log-based metrics. Interval velocities extracted from VSP data are used as an estimated vector ($m_{estimate}$), and interval velocity derived from the well log data is used as a true vector ($m_{true}$) in Equations (3)–(5). The lower values of MAPE_k and NRMSD_k, and the higher value of the R2_k score represent better data quality. The distributions for MAPE_k and NRMSD_k are quite similar, as well as R2_k, taking into account the lower MAPE_k, and NRMSD_k values correspond to higher R2_k score. The mean values depend on the exact way of the metric calculation, from log preconditioning to the first break peaking algorithm and interval velocity inversion. Hence, the main benefit comes from the relative comparison of the metrics for various experiments. The cross plots verify that all log-based kinematic metrics are related to each other, and one can use any of them for further evaluation of datasets. In our case, we chose the R2_k score as it can be naturally compared with the SNR metrics, as both the R2_k score and SNR_k should increase with increasing data quality. As for GL, the increase of GL does not correspond to better data quality based on the kinematic log-based metric. The best results are achieved for GL 4 m, followed by GL 8 m and GL 16 m. Such an observation makes sense because the smallest GL of 4 m allows robust estimation of kinematics, whereas increased GL leads to lower vertical resolution deviating from resolved log-based reference.

Figure 5c indicates the same plots for the dynamic (amplitude) metrics. In this case, the normalized maximum amplitude of the incident P-wave derived from seismic data is used as an estimated vector ($m_{estimate}$). The normalized maximum amplitude of the incident P-wave is calculated from a synthetic seismogram generated from upscaled log data and is used as a true vector ($m_{true}$). Here we can derive similar conclusions as for kinematic metrics regarding the interconnection between different metrics. We observed a significant correlation between MAPE_a and NRMSD_a, and a near-perfect inverse correlation between MAPE_a and R2_a as well as NRMSD_a and R2_a. Again, we will choose R2_a for further analysis. In contrast to kinematic metrics, we observe the increase of the data quality in terms of amplitudes with increasing GL. Such behavior is consistent with an increase in data-based SNR metrics with GL and suggests that DAS data with smaller GL may record amplitudes with lower fidelity. Finally, Figure 5d represents the cross-comparison between SNR_S, R2_k, and R2_a metrics. We can observe that all distributions in this comparison are quite different, and no coupling between the metrics is observed.

**Figure 6:**
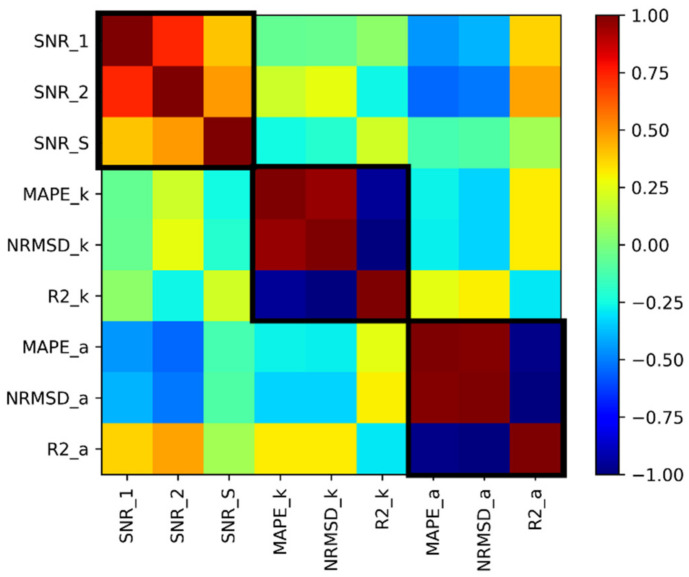
The matrix representing Pearson correlation coefficients between various calculated metrics. Correlations between data-based, kinematic, and dynamic groups of metrics are highlighted by black squares.

Figure 6 summarizes the results presented in Figure 5 more quantitatively. We calculated the Pearson correlation coefficient between each of the nine metrics with itself and the other eight metrics using Equation (6):

(6) $$R_{Pearson}\left(M_{k},M_{l}\right)=\frac{\left(M_{k}-{\bar{M}}_{k}\right)\left(M_{l}-{\bar{M}}_{l}\right)}{\left\|M_{k}-{\bar{M}}_{k}\|\|M_{l}-{\bar{M}}_{l}\right\|},$$

where $M_{k}$ and $M_{l}$ are 144-component vectors of calculated metrics for the discussed subset of 144 records; k and l are indexing metric types ($k,l\in \left[1\ldots 9\right])$. A total of 81 Pearson coefficients are calculated. The diagonal elements are equal to 1, as they represent the autocorrelation. Off-diagonal elements are color-coded by the Pearson correlation coefficient between two chosen metrics. The SNR metrics are decoupled both with kinematic and dynamic ones. Some correlation ($\left|R_{Pearson}\right|\approx 0.5$) is observed between SNR (SNR_1 and SNR_2) and dynamic metrics, as both SNR and dynamic metrics rely on the fidelity of the seismic signal amplitudes. There is a very weak ($\left|R_{Pearson}\right|<0.3$) correlation between SNR and kinematic metrics.

Within SNR metrics, we see a high correlation between SNR_1 and SNR_2 ($R_{Pearson}>0.7$) and some correlation between these two SNRs with SNR_S ($R_{Pearson}\approx 0.5$). This indicates the importance of a detailed explanation of the SNR calculation procedure in any paper discussing SNR calculations for reproducibility and comparison purposes. SNR_2 has the highest absolute correlation values ($\left|R_{Pearson}\right|≃0.5)$ for all amplitude quality metrics. Kinematic metrics, which are the primary VSP product used in the field, are not well correlated with the SNR values. Interestingly, SNR_S has a negative correlation with MAPE_k and NRMSD_k and a positive correlation with R2_k, which is the expected behavior as, with increasing, the SNR log-based metrics should improve (i.e., MAPE and NRMSD decrease, while R2 increases). SNR_2 on the other hand has a negative correlation with R2_k, which suggests that SNR in many cases can improve while the main VSP product (time curve) becomes worse (less resolved).

As we discussed before, we see a very high correlation between MAPE, NRMS, and R2 metrics separately for kinematic and dynamic cases ($\left|R_{Pearson}\right|>0.8$), which are highlighted by black squares. Hence, one can use any of the log-based metrics for the acquired data quality assessment. The most significant decoupling occurs between kinematic and dynamic-based metrics ($\left|R_{Pearson}\right|<0.3$). Next, we will analyze this decoupling in more detail to understand the influence of specific DAS data acquisition parameters.

### 3.2. Analysis of DAS Parameters

**Figure 7:**
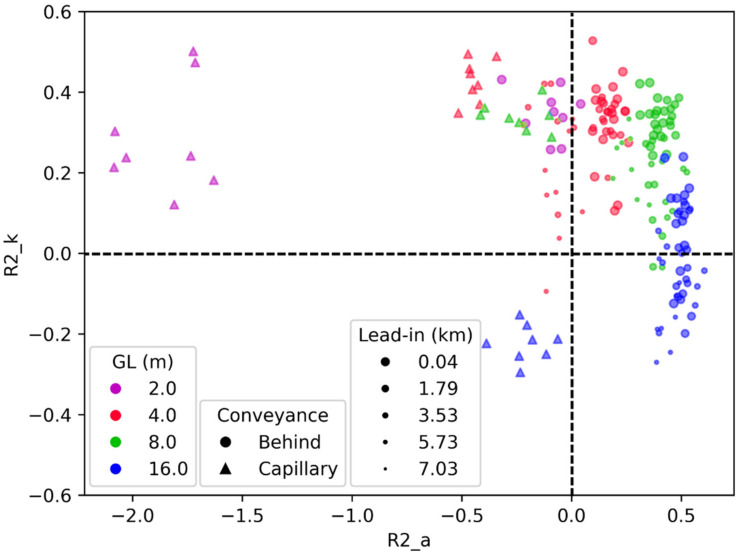
Cross-plot between kinematic (R2_k) and dynamic (R2_a) log-based metrics. Different GL coded by color, type of installation by marker type, and lead-in length by marker size. R2 values less than zero correspond to estimates that are worse than just a constant equal to correct mean values (marked by dashed lines).

To analyze how different DAS parameters influence the quality of the data in terms of inverted velocity (kinematic) and amplitudes (dynamic), we show a detailed R2_k vs. R2_a cross plot in Figure 7. This plot includes data from all 220 records, which fits into the presented limits of R2 values. Twenty-eight data points corresponding to 2 m GL and four longest lead-in values have R2 values out of the presented range. We confirm that smaller lead-in lengths (larger dot size) for behind the casing installation generally provide better results for both metrics within a fixed GL. However, kinematic metrics are affected less than dynamic ones. The response of the data quality to different GLs is the same as discussed in the previous section, now including 2 m GL. The best kinematic score is achieved with 4 m and 8 m GLs, while the best dynamic with a 16 m GL. If both kinematic and dynamic properties are essential for the survey, an 8 m GL is the optimal choice, as both metrics are relatively high. Another critical observation is related to the influence of installation type on our metrics. The capillary installation (indicated in triangles) shows excellent results for kinematic metrics (in some experiments even better than behind the casing installation for the same GL and lead-in parameters), which can be attributed to the usage of an engineered fiber in the capillary cable compared with the regular fiber used for behind the casing installation. However, the amplitude metrics are poor due to the suboptimal coupling between the fiber and the formation, which appears much worse in the case of fiber installation inside the capillary tube (control line).

## 4. Discussion

Data-based SNR metrics calculated in time or frequency domains depend on various hyperparameters (e.g., window size in time and space, window orientation, window location). SNR_1 and SNR_2 metrics, which use different noise windows, have various mean values but have high correlation coefficients. The mean values are different because the noise window for the SNR_1 metrics is affected by the sidelobes of the correlation with the sweep function. While the mean values of spectral SNR_S and SNR_2 are similar (about 25 dB), they do not have a high correlation coefficient between each other. This could be explained by differences in windows used; as for spectral SNR calculation, we need a larger time window for the spectral calculation. Additionally, a spectral SNR does not include frequencies that were not generated by a source. In cases when well log data is not available, the spectral SNR remains a suitable metric. Since it explicitly uses start and end frequencies generated by the source, spectral SNR may more accurately reflect the characteritics of real data. 

We do not observe a significant correlation between data-based and kinematic log-based metrics. This could be explained by the fact that detecting first break picks is not directly related to SNR beyond a certain threshold of detection. Moreover, the jitter noise of an interrogator unit does not influence the SNR, which can significantly affect the results of interval velocity inversion, and related log-based metrics. Note that the metrics depend on the interrogator type and used optoelectronic design, hence the proposed metrics can be used for interrogator comparison. When well log data is available, the log-based metrics are favorable to use, as we compute a new definition of “signal” based on “ground truth proxy” (log data). It is worth mentioning that sonic logging data can also become distorted by various factors, for examples borehole washouts. Therefore, data quality control is essential before log data is used as an input for metrics calculation. Furthermore, it is possible to explore metrics’ dependence on the scale by modifying chosen intervals in the data analysis workflow.

For optimizing the survey evaluation design, it is important to define the objective function or decision tree to determine the value of information [22]. Suggested metrics can serve as a decision-guiding objective function. It is important to determine the primary goal of a survey to choose a particular metric correctly. The data quality is directly related to the value of a specific metric. For example, if the survey purpose of the DAS VSP survey is to acquire a low-resolution velocity model to tie well log data with surface seismic, one can choose a log-based kinematic metric with a properly set scale hyperparameter (the parameter was set to 10 m in our case, see Figure 2). This survey can be acquired with a cost-effective fiber installation as the detection of the signal does not require perfect coupling for precise amplitude extraction. On the other hand, if the primary goal of the survey is to analyze amplitudes [23,24,25] or conduct full wavefield inversion with resolution enhancement, reliable amplitude information is crucial, and one should choose a log-based amplitude metric. 

The observed decoupling of data-based SNR metrics with kinematic metrics suggests revising common practices of SNR improvement. The results indicate that alternative conveyance options (e.g., fibers pumped into the pre-existing control line) can result in similar high log-based metrics, having significantly lower SNR values compared to when cemented behind the casing installations. Further research will target understanding the importance of the derived metrics for time-lapse acquisition and the influence of data stacking on log-based metrics.

## 5. Conclusions

We considered several metrics to quantify DAS VSP data quality. We showed that data-based and log-based kinematic and dynamic metrics are decoupled. Data-based SNR metrics provide a quick and robust way to evaluate DAS VSP data quality, while log-based metrics give more insights on fit-for-purpose acquisition optimization of parameters. Log-based metrics highly depend on additional processing steps, such as precise depth calibration, log preconditioning, and interval-velocity inversion. However, log-based metrics are necessary for advanced survey evaluation design to determine the value of an acquisition parameter and cost optimization. 

## Figures and Tables

**Table 1:** Summary of data acquisition parameters.

**Conveyance**	Behind the casing																				Capillary			
**Lead-in (km)**	0.04				1.79				3.53				5.73				7.03				0.04			
**GL (m)**	2	4	8	16	2	4	8	16	2	4	8	16	2	4	8	16	2	4	8	16	2	4	8	16
**# of records**	16	16	16	16	8	8	8	8	8	8	8	8	8	8	8	8	4	8	8	8	8	8	8	8

## Data Availability

Data and software used here are proprietary and cannot be released.
